# Determinants of favourable opinions about euthanasia in a sample of French physicians

**DOI:** 10.1186/s12904-015-0055-6

**Published:** 2015-11-05

**Authors:** Lionel Dany, Karine Baumstarck, Eric Dudoit, Florence Duffaud, Pascal Auquier, Sébastien Salas

**Affiliations:** APHM, Timone, Service d’Oncologie Médicale, 13385 Marseille, France; LPS EA849, Aix-Marseille Université, 13621 Aix-en-Provence, France; Unité d’Aide Méthodologique à la Recherche Clinique et Epidémiologique, AP-HM, Marseille, France; CRO2, Aix Marseille Université, 13284 Marseille, France; INSERM U911, 13005 Marseille, France; EA3279, Self-perceived Health Assessment Research Unit, Aix Marseille Université, 27 bd Jean Moulin, Marseille, cedex 05 F-13385 France

**Keywords:** Euthanasia, Opinions, Physicians

## Abstract

**Background:**

The question whether euthanasia should be legalised has led to substantial public debate in France. The objective of this study in a sample of French physicians was to establish the potential determinants of a favourable opinion about euthanasia in general and when faced with a specific situation as embodied in the Humbert affair.

**Methods:**

The study was a cross-sectional survey investigating two different samples of medical doctors: (1) those specialised in palliative care and affiliated to the French Society for Patient Accompaniment and Palliative Care; (2) medical interns (medical doctors in training course) in a French medical university (Marseille). A questionnaire was sent (email) to each voluntary participant including sociodemographics, professional status, mention of believing in God, and opinion about euthanasia (the question was designed to assess the general opinion about euthanasia and the opinion about a specific case, the Vincent Humbert’ case (a man who was rendered quadriplegic, blind, and mute after an accident and has requested euthanasia).

**Results:**

A total of 413 physicians participated in the research (participation rate: 48.5 %). Less than half of the population were favourable to euthanasia in general and almost two-thirds of the population were favourable to Vincent Humbert’s request for euthanasia. Based on the multivariate analysis, individuals believing in God and being a medical intern were significant independent factors linked to having a favourable opinion about euthanasia in general and about the Vincent Humbert’s request.

**Discussion:**

There is still no study in France on the development of opinion about euthanasia and its impact. The issue goes beyond the strictly professional sphere and involves broader socio-political stakes. These stakes do not necessarily take into account medical practices and experiences or the desires of end-of-life patients. The professional upheaval that the future French legal framework will doubtlessly trigger will require further research.

**Conclusion:**

The professional upheaval that the future French legal framework will doubtlessly trigger will require further research.

## Background

The issue of the legalization of euthanasia has kindled substantial public debate in several developed countries [1, 2] and particularly in France [3]. In France, a law (also called ‘the Leonetti law’, April 22nd 2005) concerning the rights of patients at the end of life allows the limitation or discontinuation of treatment and sedation for a symptom that has remained refractory until death, thereby differentiating such situations from euthanasia [4]. While euthanasia remains prohibited, much of the French population is in favour of the legalization of euthanasia. A recent poll (October 2014) conducted by a large French polling agency (Institut français d’opinion publique, IFOP) [5] found 96 % of French people to be in favour of doctors “putting an end, without suffering, to the lives of persons with an unbearable and incurable illness, if they so wish”.

To take these opinions into consideration and with a view to modifying the present law in France, several official reports and opinion surveys about the decriminalization of euthanasia or physician-assisted suicide have been requested by the French government. As highlighted by a previous report of the French National Ethics Advisory Committee (CCNE), most of those calling for the decriminalization of euthanasia or physician-assisted suicide do so in response to specific intolerable situations they have seen afflicting their relatives at the end of life [6]. In practice, it seems that approximately 9 % of terminally ill patients have expressed the wish to die but only few patients repeat their request for euthanasia, and that such requests are most often related to uncontrolled symptoms [7].

In France as in other countries [8–10], public debate and viewpoints concerning euthanasia are constantly fuelled by individual or specific situations (e.g., people with severe disability, in a vegetative state or in an end-of-life situation). Owing to their mediatization, such situations have become a key component in the debates and controversies associated with euthanasia. Indeed, our perceptions and judgments about various aspects of our environment are influenced by the process of exemplification that the media create [11]. In the last few years, a specific case in France has led to much comment and debate in society concerning the request for euthanasia. Vincent Humbert (1981–2003) was a young man who was rendered quadriplegic, blind and mute after a serious road accident. In 2002 and with the assistance of an educator, he wrote a letter to the French President (Jacques Chirac) to ask him to be given the “right to die”. Because the request was not granted, his mother assisted by a doctor “helped” Vincent to die in September 2003. The Humbert affair led the French Government to set up a parliamentary mission to examine accompaniment at the end of life and the Leonetti law.

Performing euthanasia is considered an act that only the medical profession can carry out so it is important to investigate to what extent the medical profession accepts it. This context and the stakes for health care professionals led us to investigate the opinion of different types of physicians about this issue.

International professional acceptance of euthanasia varies widely [12–18]. We can observe differences in opinions on euthanasia and assisted suicide between physicians (less favourable), other healthcare professionals and general public (more liberal). Among health professionals, being religious is generally associated with a negative attitude towards euthanasia [12, 19, 20]. Moreover, more the physicians are involved in palliative care procedures (professional practices, training), less they are favourable to the legalization of euthanasia [13, 17, 18]. Additionally, the opinions on euthanasia and physician-assisted suicide are influenced by the suffering situations on which people must express their opinions [14, 17]. The legal context also influences opinions expressed toward euthanasia. For example, physicians in Belgium and The Netherlands (countries where euthanasia is legal) are more favourable to euthanasia [12].

The objectives of this study in a sample of French physicians were to establish the potential determinants of a favourable opinion about euthanasia: i) in general and ii) when faced with a specific situation as embodied in the Humbert affair.

## Methods

### Design

The study was a cross-sectional survey investigating two different samples of medical doctors: (1) those specialised in palliative care and affiliated to the French Society for Patient Accompaniment and Palliative Care; (2) medical interns (a ‘medical intern’ corresponds to a medical doctor in training course; after the sixth year of medical studies, the medical intern practices in the hospital, provides medical treatment or performs surgery under the supervision of a senior doctor) of a French medical university (Marseille, South of France) who are in their 7th and 8th years of the training course. Mailing lists were provided by the French Society for Patient Accompaniment and Palliative Care and the university. The exclusion criterion was refusal to participate. A questionnaire including the following data was sent via e-mail to all eligible individuals: i) sociodemographics (sex, age, marital status, children); ii) information relative to professional status (supplementary specialization, palliative care experience); iii) one item relative to believing in God (‘Do you believe in God?’ yes/no) and one item concerning the history of caring for someone in a palliative care situation; iv) opinion about euthanasia using a 6-point scale encoded from 1 strongly disagree to 6 strongly agree; the question was designed to assess the general opinion about euthanasia and to know what the subjects thought about the case of Vincent Humbert. French euthanasia law defines euthanasia as the intentional ending of a life by the administration of medication by a physician at the explicit request of a patient. This is the definition that has been used in this work. Physician-assisted suicide is the practice of providing a competent patient with a prescription for medication for the patient to use with the primary intention of ending his or her own life. French law considers euthanasia as illegal and subject of a criminal penalty. French law does not use the term “physician-assisted suicide”.

### Ethics

The study conformed to the principles of the Declaration of Helsinki and French Good Clinical Practices. This present study is defined as an non-interventional research as mentioned in articles L.1121-41 and R.1121-2 of the French Public Health code: it does not involve products mentioned in article L.5311-1 of the French Code of Public Health and does not imply any changes in the medical care received by the patients. According to the French law (Article L1121-1, Law n°2011–2012 29 december 2011-art. 5), ethical approval is not needed. All subjects participated voluntary. All participants were informed about the objectives and the design of the research and participated after giving their consent. Anonymity and confidentiality were maintained during the survey.

### Statistical analysis

The data are expressed as frequencies and percentages for categorical variables. Euthanasia opinions (general opinion and opinion about Vincent Humbert) initially collected from the 6-point scale were re-coded in a binary variable [17]: scores from 1 to 3 represented an unfavourable opinion and scores from 4 to 6 were considered favourable. Comparisons between the favourable and unfavourable groups were performed using the chi-square or Fisher’s exact tests for frequencies. Multivariate analysis using logistic regression models were performed using a forward stepwise approach to determine variables potentially linked to a favourable opinion. Variables relevant to the models were selected on their clinical interest and/or a threshold *p*-value ≤0.2 in univariate analysis (gender was systematically entered into the model). The final models expressed the odds ratios and 95 % confidence intervals. All the tests were two-sided. Statistical significance was defined as *p* < 0.05. Statistical analyses were performed using the SPSS version 19.0 software package (SPSS Inc., Chicago, IL, USA).

## Results

### Population

In total, 413 physicians participated in the research. The participation rate was 48.1 and 48.6 % for medical doctors specialised in palliative care and training course medical doctors respectively. The socio-demographic and professional characteristics of the sample are summarized in Table 1. The mean age was 34.3 years (standard deviation SD: 11.1) and the sex ratio was 0.73. Half of them declared they believe in God and the same proportion had taken care of someone nearing the end of life. Nearly six out of 10 physicians were medical interns and more than two-thirds were specialised in general practice.

**Table 1:** Characteristics of the sample

	Number	Percent
Gender		
Female	239	57.9
Male	174	42.1
Age		
≤ 27 years	215	52.1
> 27 years	198	47.9
Marital status		
Couple	281	68.0
Single	132	32.0
Child		
Yes	163	39.5
No	250	60.5
Believes in God		
Yes	210	50.8
No	203	49.2
Has taken care of someone nearing the end of life		
Yes	203	49.2
No	210	50.8
Professional status		
Palliative care specialists	170	41.2
Medical interns	243	58.8
Specialisation		
General practice	283	68.5
Others ^a^	130	31.5
Opinion about euthanasia		
Unfavourable	232	56.2
Favourable	181	43.8
Opinion about the demand of euthanasia of Vincent Humbert		
Unfavourable	167	40.4
Favourable	246	59.6

^a^Others: oncology, surgery, anaesthesia, psychiatry, pneumology, public health, gynaecology

**Table 2:** Opinion about euthanasia in general

		Opinion			Multiple regression^b^		
		Unfavourable (1)	Favourable (2)	*p* ^a^	OR	IC (95 %)	*p*
		n (%)	n (%)		(1) versus (2)		
Gender	Female (*n* = 239)	140 (58.6)	99 (41.4)	0.146	1	[0.74–2.25]	0.353
	Male (*n* = 174)	92 (52.9)	82 (47.1)		1.29		
Age	>27 years (*n* = 198)	172 (86.9)	26 (13.1)	<0.001	1	[0.42–2.55]	0.924
	≤27 years (*n* = 215)	60 (27.9)	155 (72.1)		1.04		
Marital status	Couple (*n* = 281)	179 (63.7)	102 (36.3)	<0.001	1	[0.47–1.48]	0.539
	Single (*n* = 132)	53 (40.2)	79 (59.8)		0.83		
Child	Yes (*n* = 163)	151 (92.6)	12 (7.4)	<0.001	1	[0.47–4.73]	0.493
	No (*n* = 250)	81 (32.4)	169 (67.6)		1.49		
Believes in God	No (*n* = 203)	119 (58.6)	84 (41.4)	0.188	1	[1.08–3.26]	0.024
	Yes (*n* = 210)	113 (53.8)	97 (46.2)		1.88		
Has taken care of someone nearing the end of life	Yes (*n* = 203)	133 (66.0)	68 (34.0)	<0.001	1	[0.72–2.15]	0.429
	No (*n* = 210)	98 (46.7)	112 (53.3)		1.24		
Professional status	Pall care specialists (*n* = 170)	164 (96.5)	6 (3.5)	<0.001	1	[13.18–248.22]	<0.001
	Medical interns (*n* = 243)	68 (28.0)	175 (72.0)		57.20		
Specialisation	Others (*n* = 130)^c^	84 (64.6)	46 (35.4)	0.012	1	[0.43–1.54]	0.548
	General practice (*n* = 283)	148 (52.3)	135 (47.7)		0.82		

^a^ *p*-Value for Pearson’s χ^2^

^b^Hosmer-Lemeshow test: *p* = 0.316

^c^Others: oncology, surgery, anaesthesia, psychiatry, pneumology, public health, gynaecology

**Table 3:** Opinion about Vincent Humbert’s request for euthanasia

		Opinion			Multiple regression^b^		
		Unfavourable (1)	Favourable (2)	*p* ^a^	OR	IC (95 %)	*p*
		n (%)	n (%)		(1) versus (2)		
Gender	Female (*n* = 237)	94 (39.3)	145 (60.7)	0.332	1	[0.37–1.20]	0.184
	Male (*n* = 174)	73 (42.0)	101 (58.0)		0.67		
Age	>27 years (*n* = 196)	141 (71.2)	57 (28.8)	<0.001	1	[0.10–2.14]	0.330
	≤27 years (*n* = 215)	26 (12.1)	189 (87.9)		0.47		
Marital status	Couple (*n* = 281)	138 (49.1)	143 (50.9)	<0.001	1	[0.52–1.99]	0.956
	Single (*n* = 132)	29 (22.0)	103 (78.0)		1.01		
Child	Yes (*n* = 163)	127 (77.9)	36 (22.1)	<0.001	1	[0.49–3.70]	0.563
	No (*n* = 250)	40 (16.0)	210 (84.0)		1.34		
Believes in God	No (*n* = 203)	88 (43.3)	115 (56.7)	0.139	1	[1.27–4.21]	0.006
	Yes (*n* = 210)	79 (37.6)	131 (62.4)		2.31		
Has taken care of someone nearing the end of life	Yes (*n* = 203	96 (47.8)	105 (52.2)	0.002	1	[0.45–1.48]	0.512
	No (*n* = 210)	70 (33.3)	140 (66.7)		0.82		
Professional status	Pall care spec (*n* = 170)	139 (81.8)	31 (18.2)	<0.001	1	[12.63–409.22]	<0.001
	Medical interns (*n* = 243)	28 (11.5)	215 (88.5)		71.91		
Specialisation	Others (*n* = 130)^c^	63 (48.5)	67 (51.5)	0.016	1	[0.42–1.46]	0.447
	General practice (*n* = 283)	104 (36.7)	179 (63.3)		0.78		

^a^ *p*-Value for Pearson’s χ^2^

^b^Hosmer-Lemeshow test: *p* = 0.794

^c^Others: oncology, surgery, anaesthesia, psychiatry, pneumology, public health, gynaecology

### Opinion about euthanasia in general

Less than half of the population were favourable to euthanasia in general. Univariate analysis showed that younger physicians, single physicians, those without any children, those who had not taken care of someone nearing the end of life and those specialised in general practice were more favourable to euthanasia. Medical interns were significantly more favourable to euthanasia than palliative care specialists. Based on the multivariate analysis, individuals believing in God and being a medical intern were significant independent factors linked to having a favourable opinion about euthanasia in general. The results were detailed in Table 2.

### Opinion about Vincent Humbert’s request for euthanasia

Almost two-thirds of the population were favourable to Vincent Humbert’s request for euthanasia. Univariate analysis showed that younger physicians, single physicians, those without children, those who had not taken care of someone nearing the end of life, medical interns and those specialised in general practice were more favourable to Vincent Humbert’s request. Multivariate analysis showed that independent factors associated with a favourable opinion about Vincent Humbert’s request were believing in God and being a medical intern. The results were detailed in Table 3.

## Discussion

While 96 % of French people-have-been found to be in favour of euthanasia, fewer than 50 % of physicians were favorable to it. This underlines the fact that this is an important medical and social problem in France, as it also is worldwide. Euthanasia is defined by the law in only three countries in Europe [16], we decided to study the potential determinants of being favourable to euthanasia in a sample of French physicians. One of the most important findings in this study is the difference between the two populations of physicians concerning their opinion about euthanasia. Most of the medical students were in favour of it whereas palliative care specialists were largely opposed. In this sense, medical interns seem to have opinion close to that of the French population [5]. In addition, while physicians were unfavourable to euthanasia, their opinions were not as categorical when they were faced with the specific case of Vincent Humbert. Physicians, and specifically palliative care specialists, presumably took a “principled stance” linked to their professional identity when they express their opinion about euthanasia in general. When they were faced with a specific case, their stance was nuanced because they were confronted with a different level of personal involvement. Their opinions may have varied depending on the clinical situation. They were even more in favour of euthanasia since such patients are symptomatic and have a major handicap while most patients in palliative care are cancer patients who may be symptomatic but remain valid.

The medical interns are younger and less (or not) experienced concerning end-of-life and palliative care. Research has shown that the physicians most heavily involved in palliative care (training, management of patients at the end of life) are the least in favour of the legalisation of euthanasia, are more aware of the aims of palliative care, and are in favour of the professionalization of this medical speciality [13, 17, 18]. Furthermore, the WHO definition of palliative care, like the definition given by the French Society of Accompaniment and Palliative Care (Société Française d'Accompagnement et de soins Palliatifs, SFAP), specifies that the aim of palliative care ‘is neither to hasten nor postpone death’ [21, 22]. In other words, the opinions expressed toward euthanasia by our sample highlight the professional and personal stakes involved. For palliative care professionals, opposition to euthanasia is particularly important in order to maintain their professional legitimacy [18]. As pointed out by others, the relationship between palliative care and euthanasia comprises semantic and strategic issues related to the professionalization of palliative care. For example, attitudes toward the professionalization of palliative care and proximity with its providers influence beliefs about which medical practices should be labelled as euthanasia [17].

Another explanation that may explain the distance that palliative care specialists take regarding the issue, is that only few terminally ill patients express a wish to die or repeatedly request euthanasia [6]. In fact, the question of euthanasia is not central to their professional practice and the relatively rareness of such requests from patients may distance them from it. Finally, the difference in opinion between palliative care specialists and medical interns could be analysed in terms of a generational difference since the on-going social debate in France in recent years may have served to change the way future physicians regard this issue in general.

Promoting palliative care and advocating the legalization of voluntary euthanasia and assisted suicide (VE/AS) are generally viewed as opposite causes [23]. Both positions represent traditionally opposed currents of thought but also reflect concern for many common values (i.e. reducing human suffering; not reducing patients to a purely vegetative state; empowering patients at the end of life) [23]. Promoting the development of palliative care is often put forward as an alternative in debates on mooted changes to laws concerning the end of life, with the risk of reinforcing opposition between palliative care organizations and advocates of euthanasia.

Differences in opinion toward euthanasia may not be related only to professional identity or professional stakes. In our study, believing in God was associated with a more favourable opinion about it. This finding differs from others that demonstrate a link between religiosity and opposition toward euthanasia. Indeed, the independent effect of religiosity has been extensively discussed in previous studies [19, 20]. Beyond religious affiliation, strong religiosity has an impact on opinion about legalization of euthanasia [19, 20]. For example, supporting such legalization was less frequent among physicians attending religious services at least once a month [13]. In other study we showed that stronger religious belief was associated with more positive support for euthanasia [24]. However, the measures used to assess religiosity in these studies were not the same. Some authors built a religiosity index including different items, as “believing in God?” for example [25]. Other studies used more specific questions as the “religious affiliation” [15], the “belief in the church’s teachings” [16], or the “attendance to religious services” [17]. In our study, the association between “believing in God” and a more favourable opinion toward euthanasia can be explained by the specificity of the used question. But considering that “belief in God” constitutes a quasi-philosophical thinking, does not imply, for people, a strong religiosity or the adherence to strict dogma. In our study, we did not ask the respondents for their religious affiliation because surveys in France are seldom allowed to ask such questions. In addition, we did not ask a more general question about spirituality because the term ‘spirituality’ in French has several meanings. Furthermore we did not assess the level of religiosity but a more general belief concerning God. People who declare they believe in God are able express nuanced positions about key issues such as the “sanctity of life”, patient autonomy and other ethical issues.

The implementation of palliative care is closely linked to considerations within society concerning conditions at the end of life and euthanasia. This issue is frequently addressed in the specialist literature on palliative care [26]. While most of the French population is in favour of euthanasia, it is not authorised under French law. Our results are important because the issue is very topical at present within French society. The French National Assembly recently proposed legislation (17 March 2015) concerning the end of life and adopted at the first reading a bill that establishes a right to “deep continuous sedation” for end-of-life patients and makes binding any advance care that the patient may plan. Future modifications to legislation in France concerning the end of life will likely accentuate the debate concerning the specificities of palliative care practices and their ethical and identity issues. Palliative care specialists could be brought to question their opposition to euthanasia and the extent to which they can remain neutral on the issue [27].

We arbitrary decided to consider the euthanasia opinion as a binary variable, with the recoding from the 6-point scale, leading to a loss of information and a reduction of the statistical power to detect a relation between the variable and patient outcome. However, our choice remains on three main arguments. This approach: i) is already used in similar work [17, 28], ii) leads to easier interpretation and presentation of results, and iii) permits comparisons in the French context.

## Conclusion

There is still no study in France on the development of opinion about euthanasia and its impact. The debate related to euthanasia probably exceeds the socio-political stakes and also concerns the healthcare professional sphere. In addition, these stakes do not necessarily take into account medical practices and experiences or the desires of end-of-life patients. The professional upheaval that the future French legal framework will doubtlessly trigger will require further research.
